# ACHIEVING EQUITABLE QUALITY OF CARE FOR NURSING HOME RESIDENTS

**DOI:** 10.1093/geroni/igac059.230

**Published:** 2022-12-20

**Authors:** Jasmine Travers

**Affiliations:** NYU, New York City, New York, United States

## Abstract

Inequities in care delivery within nursing homes are pervasive across the United States. Racial and ethnic minority residents disproportionately reside in nursing homes of poorer quality, lower staffing, and fewer resources when compared to their White counterparts. Dr. Jasmine Travers, a health services researcher at New York University and with expertise in disparities and long-term care, will present on recommendations to achieving equitable quality of care for nursing home residents.

